# Fused Multi-Domains and Adaptive Variational Mode Decomposition ECG Feature Extraction for Lightweight Bio-Inspired Key Generation and Encryption

**DOI:** 10.3390/s24247926

**Published:** 2024-12-11

**Authors:** Israel Edem Agbehadji, Richard C. Millham, Emmanuel Freeman, Wanqing Wu, Xianbin Zhang

**Affiliations:** 1 Honorary Research Fellow, Faculty of Accounting and Informatics, Durban University of Technology, P.O. Box 1334, Durban 4000, South Africa; 2 ICT and Society Research Group, Department of Information Technology, Durban University of Technology, P.O. Box 1334, Durban 4000, South Africa; 3 Centre for Augmented Intelligence and Data Science, School of Computing, University of South Africa, Johannesburg 1709, South Africa; efreeman@gctu.edu.gh; 4 School of Biomedical Engineering, Sun Yat-sen University, Guangzhou 510275, China; wuwanqing@mail.sysu.edu.cn (W.W.); zhangxb55@mail2.sysu.edu.cn (X.Z.)

**Keywords:** time-domain feature extraction, lightweight encryption, adaptive variational mode decomposition, ECG feature extraction, bio-inspired key generation

## Abstract

Security is one of the increasingly significant issues given advancements in technology that harness data from multiple devices such as the internet of medical devices. While protecting data from unauthorized user access, several techniques are used including fingerprints, passwords, and others. One of the techniques that has attracted much attention is the use of human features, which has proven to be most effective because of the difficulties in impersonating human-related features. An example of a human-related attribute includes the electrical signal generated from the heart, mostly referred to as an Electrocardiogram (ECG) signal. The methods to extract features from ECG signals are time domain-based; however, the challenge with relying only on the time-domain or frequency-domain method is the inability to capture the intra-leading relationship of Variational Mode Decomposition signals. In this research, fusing multiple domains ECG feature and adaptive Variational Mode Decomposition approaches are utilized to mitigate the challenge of losing the intra-leading correlations of mode decompositions, which might reduce the robustness of encryption algorithms. The features extracted using the reconstructed signal have a mean (0.0004), standard deviation (0.0391), skewness (0.1562), and kurtosis (1.2205). Among the lightweight encryption methods considered, Chacha20 has a total execution time of 27µs. The study proposes a lightweight encryption technique based on the fused vector representation of extracted features to provide an encryption scheme in addition to a bio-inspired key generation technique for data encryption.

## 1. Introduction

Feature extraction is one of the topical issues in medical diagnosing. In order to have a proper diagnosis, one needs to understand the nature of feature extraction and its role in determining a diagnosis. The typical features used in a medical diagnosis include an Electroencephalogram or Electrocardiogram; while many techniques have been applied to feature extraction in the context of an Electrocardiogram (ECG), the effectiveness of these techniques has constantly been enhanced. Research has proven that using human features to create an encryption algorithm provides a much stronger approach notwithstanding the anticipated challenge it may have. Nonetheless, human biometric information are a promising alternative for cryptographic key generation to the traditional use of passwords as cryptographic keys. Cryptographic keys generated from biometrics are difficult to forge and thus expand the frontiers of research into the use of different biometric key generation approaches. Keys can be extracted from biometric features, in raw ECG signals, which can be reliable despite the noise in ECG signals or abnormalities [1]. ECG-based biometric systems are much more reliable than other present biometric systems, e.g., fingerprints [2]. Furthermore, the widespread adoption of ECG technology in some clinical or hospital facilities and the ease of deploying ECG sensors in consumer settings in wearable devices [3] makes ECG signal encryption more imperative. Security-related application domains, when equipped with ECG signal encryption, provide many strong features to secure data on devices.

Currently, most Internet of Things (IoT) devices are championing the use of human features in encrypting data from medical devices. These IoT devices used in the context of medical diagnosing are so small that using traditional encryption algorithms on these devices may require more energy for computation [4]. Though most consider the use of a cloud computing framework to provide the needed encryption, it also requires that devices are constantly connected to the internet for data encryption [5]. The efficiency and security for privacy are often very distinct leading to the proposition of an image privacy protection scheme that ensures high-quality reconstruction using the discrete cosine transform compression and nonlinear dynamics [6]. When IoT devices use ECG signals for feature extraction, then the amplitude and intervals are used, which are further processed [7]. Here, it is the case that IoT devices are used to capture and monitor ECG features. It is very common these days to have wearable ECG devices attached to the human skin to continuously monitor ECG signals. This development demonstrates how IoT devices have evolved. Again, it is common to have IoT devices and smart watches equipped with ECG sensors to monitor a person’s heartbeat. The integration of IoT in these areas suggests easy-to-collect ECG features; however, the challenge is the computation algorithm to ensure encryption using ECG features. Therefore, crafting any algorithm based on an ECG signal should be very lightweight. One of the key phases in the integration of IoT with ECG is the security of data being transmitted.

Feature extraction in the context of an ECG requires the identification and analysis of features in ECG data. When dealing with ECG feature extraction, the amplitude and intervals are very time-dependent. Thus, time-domain feature extraction may have to deal with the intra-leading relationship within the sequence of multiple ECG signals from the same person. Mostly, statistical and machine learning techniques play a leading role in analyzing these intra-leading correlations and thus model accuracy in analyzing these is imperative. Again, these intra-leading relationships are key for a comprehensive understanding of the heart’s electrical activity and for making accurate diagnoses based on ECG data. One will expect that while these electrical activities are happening, the approach to encrypting these data should be robust, taking into account the intra-leading correlations. This research seeks to develop a lightweight encryption algorithm that considers these intra-leading relationships using a time-domain feature, frequency-time domain, and adaptive variational model decomposition-based technique. Existing cryptographic schemes are complex, such that the key generation consumes a large computation time and a large amount of energy [8]; thus, encryption schemes need a suitable lightweight approach to encryption. Thus, this study contributes to the introduction bio-inspired algorithm based on the Kestrel-based search algorithm as it randomizes searching with its half-life component, which adds layers of randomization for a more secure key generation for encryption. The advantage of the Kestrel-based approach is the ease of formulation, which does not add more computational cost in the search for an optimal key.

The remainder section is organized as follows: Section 2 (literature review), Section 3 (methods and materials), Section 4 (results), Section 5 (discussion), and the conclusion in Section 6.

## 2. Literature Review

This section focused on reviewing articles on multiple domain feature extraction techniques and also lightweight encryption mechanisms. The review is necessary to know what has been performed and the gap that needs to be filled through our research. These sections are aligned with the research topic, thus helping the crystallization of articles along the thematic domains.

### 2.1. Model-Based and Multi-Fusion Domain Bio-Signals Techniques

ECG signals are electrical impulses generated by the heart’s activity, and these signals are recorded in series of waves (P, Q, R, S, T), representing the varied phases of the electrical activities of the heart’s cycle from its starts (P wave) to the end of the sequence (T wave). The intermediate waves are the time to transition from one wave to another wave. Thus, the regular and timing features are very relevant in diagnosing any heart-related condition. In this regard, the ECG is very crucial in diagnosing heart-related issues. To capture these electrical activities, electrodes are placed on the skin of an individual and then the waves are monitored, which helps to differentiate people based on these waves [2].

The time-domain approach quantifies changes in ECG signal over time. Among the time domain features include average heart rate variations, R-R intervals, and Shannon entropy [9,10].

Zhao, Li [11] segmented and extracted ECG features into a time-domain matrix. Then, the periodic signal was transformed into a wavelet to output the frequency domain features in the matrix structure. Furthermore, a nature-inspired algorithm such as particle swarm optimization (PSO) was utilized to fine-tune parameters to optimize the extraction of ECG features. To address the accuracy of ECG feature identification from different domains, such as time, frequency, or time–frequency; the multi-feature fusion method was proposed, which combined Variational Mode Decomposition (VMD) and the Convolutional Neural Network (CNN) [12]. On one hand, the VMD technique was used for feature decomposition while the CNN was used to extract feature information from the ECG signal. Furthermore, the features extracted were weighted and fused for ECG signal recognition. Machine learning models have been leveraged for ECG feature extraction to help in cardiac evaluation and treatment decisions [13]. It has been indicated that ECG signals are time domain-reliant, leading to the conversion to spectrogram signals using a Short-Time Fourier Transform (STFT) [14]. Thus, different signals of heartbeat were segregated into a deep learning model for training. While using an open dataset, the six best P-QRS-T fragments were extracted based on priority and the normalization of positions using the non-fiducial symlets and non-fiducial daubechies [15]. Unfortunately, the accurate identification of fiducial points is a very challenging task in ECG signals if not well addressed: it can degrade the performance of the ECG-based biometrics [16]. This leads to the proposition of a framework based on ECG signal for user authentication, which does not need the detection of fiducial points. This framework utilized data-adaptive Variational Model Decomposition for noise removal and feature extraction from the ECG signal. Pradhan, Neelappu [17] suggested that mode decomposition approaches (e.g., empirical mode decomposition) are effective in signal analysis.

Physiological signals can be linked to emotions because both provide unconscious responses, suggesting that ECG features can help recognize people’s emotions, which can influence their physiological responses at any given time [18]. The ubiquity of wearable ECG devices helps to recognize people’s emotions; however, there are high chances of ECG signal contamination, which is caused by motion artifacts, thus leading to a decline in distinguishing ECG features [19]. The feature extraction algorithm for coronary heart disease detection using photoplethysmography used three algorithms that are respiratory rate (RR) interval, HRV Features, and Time Domain Features [20]. A photoplethysmograph (PPG) is a biomedical signal capable of detecting blood volume changes in the microvascular bed of tissues [21]. Myocardial infarction, also known as heart attack, was detected using 21 time-domain features that are extracted from 12-lead ECG signals [22]. Gender classification based on ECG signals has also been proposed using time and frequency domain features [23]. The Time Multiplexed Fast Fourier Transform (TMFFT) approach was used to extract features for categorization into the frequency domain for Arrhythmia classification [24]. An Electrocardiogram (ECG) is broadly utilized for monitoring and diagnosing cardiac arrhythmia, which is an irregularity of the heartbeat that can potentially cause difficulties that create an instantaneous life risk [25]. In this regard, the Selective Opposition (SO)-based Artificial Rabbits Optimization (SOARO) strategy was applied to extract different features on time, time–frequency, entropy, and nonlinearity features of ECG [25]. In the context of the Autism Spectrum Disorders screening method, an acoustic method was employed in speech processing, where the acoustic features are constructed based on time–frequency domain independent component analysis (TF-ICA). In this approach, three methods used are, firstly extracting and combining the rows of the unmixing matrix of each frequency point to build the feature vector; secondly, entailing the separation of results on each frequency point as a time–frequency feature; and lastly, entailing the extraction of time-domain features from the outputs of TF-ICA [26]. When time-domain (TD) and frequency–time-domain (TFD) features are used together in a movement classification, it improves efficiency [27]. The Singh and Krishnan [28] approach leads to the extraction of the time domain, frequency domain, and time–frequency domain features in addition to the use of decomposition and sparse domain for ECG signal processing.

In the context of person identification, different EEG features like time domain, frequency domain, and time–frequency domain features were extracted and fused, in which a supervised learning approach was applied and evaluated in terms of accuracy rate, specificity, sensitivity, and F-score, and it was determined that the fusing method is efficient for user authentication [29].

Deep learning models such as the spatiotemporal deep learning technique have been applied to learn time-domain features, which are extracted into a matrix structure [30]. Furthermore, Khushaba, Phinyomark [31] proposed a simple time-domain feature extraction technique that leverages the capability of waveform length, zero crossings, and root mean squared to capture the relation between any number of channels.

In some instances, multiple domain feature extraction approaches were used combined with ensemble machine learning methods for classification and prediction [32]. Wavelet packet transform (WPT) and Short-Timed Fourier Transform (STFT) approaches were used to extract features from EEG signals. It has been indicated that using a single feature does not yield better performance compared to the fusion of multiple features [33]. In these regards, model-based approaches have been applied for both ECG and EEG feature extraction, and examples of such models include CNN and supervised learning. Again, fusing multiple different ECG features can provide an effective way to develop an encryption algorithm for the Internet of Medical Things. The feature normalization approach proposed used a binary classifier based on a support vector machine to classify features for high classification accuracy [34].

### 2.2. Lightweight Encryption Mechanisms

The unique properties of ECGs described in the previous section demonstrate the reason why it is preferred for user identification rather than the use of more traditional methods, such as passwords, etc. [35]. This section mainly focuses on the encryption mechanism.

Hash function and DNA cryptography were used to implement the Triple Data Encryption Standard (Triple-DES) that combines Hash function and DNA cryptography to encrypt different bio-signals into the DNA format. Mathivanan, Ganesh [36] proposed a system to convert ECG signals into QR codes. Additionally, Karthikeyan and Martin Leo Manickam [37] introduced a secret key generation algorithm extracted from the parameters of the ECG signal to allow device authentication. A reversible bio-signal steganography method was applied using the Extended Binary Golay Code based on the error correction method [38].

A wavelet-based 128-bit key generator using the uniqueness and quasi-stationary biometric behavior of ECG signals of individuals was proposed: there were two stages: key generator on enroll and verify and another on key determination with an algorithm [39]. Many encryption algorithms that rely on the key size of the 256-bit key have also been proposed, which include the Chacha20 encryption scheme [40]. This scheme encrypts data a byte at a time leading to the generation of stream cipher for data encryption [41].

Heartbeat-based Random Binary Sequences (RBSs) that generate 128-bit RBSs using inter-pulse intervals (IPIs) of heartbeats incorporate a finite monotonic increasing sequence generation mechanism of IPIs and a cyclic block encoding procedure that extracts a high number of entropic bits from each IPI [42].

The generation of a security key using the R-R interval feature of ECG signals as an input for verification and identification occurs by generating a security key corresponding to an individual. The system comprises two independent stages: registration and authentication. The biometric security key, created in the registration stage, was generated using Hamming Distance and the extended version of the triple DES algorithm. Biometric security key generation, verification, authentication, and performance of the biometric security key have been assessed using the R-R interval of ECG signals taken from the standard MIT-BIH database [43]. The simulation results for 64-bit, 128-bit, and 256-bit biometric security keys indicate that the performance of the proposed biometric security key is reasonably good for a security system.

An energy-efficient and computationally less complex authentication technique for BSN, which is a biometric-based algorithm, is proposed, which utilizes Heart Rate Variability (HRV) for a simple key generation process. The proposed algorithm is compared with three data authentication techniques, namely Physiological Signal Key Agreement (PSKA), Data Encryption Standard (DES), and Rivest Shamir Adleman (RSA). The results suggest that the proposed algorithm is quite efficient in terms of transmission time utilization, average remaining energy, and total power consumption [8]. The RSA encryption algorithm is utilized to encrypt an ECG signal; however, the RSA algorithm only performs one operation on encrypted data, which can either be addition or multiplication [44].

Generation of the fly without requiring the key pre-distribution solutions approach was proposed involving two different Interpulse Interval (IPI) features of ECG-based cryptographic key generation. The first approach is realized by using a pseudo-random number and consecutive IPI sequences. The second approach is realized by utilizing the Advanced Encryption Standard (AES) algorithm and IPI as the seed generator for the AES algorithm [45].

Due to intra-individual variability, bio-crypto keys (bio-keys), in the context of wearable devices, based on Electrocardiograms (ECGs), were proposed for flexibility and convenience to use bio-key using ECGs. This approach minimizes biosignal variability using normalization, clustering-based binarization, and the fuzzy extractor, enabling the generation of personalized seeds and offering ease of use with the accuracy of authentication [46].

Moosavi, Nigussie [45] combined two different bio-signals, such as ECG and EMG, to generate keys in cryptographic systems by initially using a “pseudo-random number” and consecutive IPI sequences, then followed by the use of an “Advanced Encryption Standard” (AES) algorithm and IPI as a seed generator for the AES algorithm. The advantage of this approach is that it avoids pre-key distribution and ensures ease of key generation. Karthikeyan and Manickam [47] proposed an authentication model for a low resource-constrained architecture where a secret key is generated from the ECG signal parameter and combined with the Secure Force (SF) algorithm in a wireless network.

The generation of a persistent key from an ECG signal to ensure symmetric encryption of data in a time-invariant key has been considered in [48]. Similarly, a time-invariant cryptographic key generation mechanism based on electroencephalogram (EEG) signals has also been proposed [49]. A key generation approach that uses a wavelet-based 128-bit key generator from ECG signals was proposed, which comprises two independent steps, that is, enrollment and verification generation [39].

The ECG signal is distinct to an individual such that it is very difficult to emulate; therefore, securing these features of ECG so that only an authenticated person can assess these signals for diagnosis purposes is imperative [50]. Furthermore, the processing of the ECG signal was achieved with the QRS complex method, which shows the heart rates (HR) that can be visually seen and traced; thus, it is easy to encrypt the visual part of ECG tracing.

ECG signals are used for identification because it varies between individuals [51]. From a diagnostic perspective, individuals who have a background of heart-related issues and have a long record of ECG require a large amount of storage space [52]. Pan and Tompkin’s algorithm helps in the detection of ECG signal [52]. In these regards, selecting the optimal key parameter is significant for an encryption algorithm and this optimization was achieved using the glow-worm swarm optimization method for encryption [5]. Thus, this suggests that a nature-inspired optimization algorithm can play a role in key generation for an encryption algorithm. ECG encryption technique relies on DNA layers and AES to reduce the encryption execution time and improve security for IoT health applications [53]. Methods to extract ECG features include the Lyapunov exponent‘s spectrum in which the extracted features are used as a secret key to encrypt pictures and text messages [54].

## 3. Materials and Methods

The method and material section outlines the stages to preprocess ECG signals, extract features, normalize features, generate the feature vector, conduct a statistical analysis on the feature vector including zero crossing, and then save the final feature vector. Developing an encryption algorithm using ECG (Electrocardiogram) signals is an intriguing idea that combines elements of biometric security with cryptography. ECG signals are unique to individuals, which means they can serve as a basis for personalized encryption. The following sub-sections detail how the study approaches this.

### 3.1. Loading of the ECG Signal Dataset

The ECG signal is loaded from a file or data source. Each recording has a 20-s single-lead ECG signal from LIMB II, with a sampling rate of 500 Hz. The dataset contains 89 ECG recordings, including 25 from healthy individuals in a lab setting, 20 from the MIT-BIH Arrhythmia Database (MITDB), and 44 from cardiac patients in a clinical environment. These data are stored in the “data. mat” file and can be read using Python 3.13 with the Scipy package. The package is organized as a dictionary, with corresponding labels and original signal data, each containing 10,000 data points. The input to the model is the BIH Arrhythmia Database (MITDB).

### 3.2. Preprocess ECG Signal

The raw ECG signal is cleaned from noise and normalized for further processing. The band-pass filter approach is used to remove noise, which is expressed as a frequency response *H(f)* (see Equation (1)). The band-pass filter allows only a specific range of frequencies to pass while attenuating frequencies outside this range [0, 1].

(1) $$H\left(f\right)=\left\{\begin{array}{c} 1\;for\;f_{L}\leq f\leq f_{H}\\ 0,\;otherwise \end{array}\right.$$

where *f* is frequency, *f_L_* is the lower cutoff frequency, and the upper cutoff frequency is *f_H_*; therefore, Equation (2) is as follows:

(2) $$h\left(t\right)=h_{LP}\left(t\right)-h_{HP}\left(t\right)$$

where $h_{LP}\left(t\right)$ and $h_{HP}\left(t\right)$ represent the impulse responses of the low-pass and high-pass filters, respectively. To improve the model’s ability to generalize, the recordings were normalized and scaled to a common range (e.g., 0 to 1) using min–max scaling expressed in Equation (3), as follows:

(3) $$F_{norm}=\frac{F-F_{min}}{F_{max}-F_{min}}$$

where $F_{min}$ and $F_{max}$ are the min and max feature normalization in the range [0, 1].

### 3.3. ECG Extract Features

The adaptive variational model decomposition (adaptive VMD) approach is used to decompose the ECG signal and further remove noise. Thus, given the ECG signal *x(t),* the decomposition is expressed in Equation (4), as follows:

(4) $$x\left(t\right)=\sum_{i=1}^{M}S_{i}\left(t\right)+n\left(t\right)$$

where *x(t)* is the observed signal, $S_{i}(t)$ is *ith* signal, n(t) is residual noise, and M is the total number of signals. Residual noise in the ECG signal and noise after ECG signal reconstruction can impact the feature extraction with the adaptive VMD; thus, functions expressed in Equations (5)–(8) were employed to address these noises.

The variational mode defines the likelihood function *Fn*, which is expressed in Equation (5), as follows:

(5) $$Fn=p\left(x\right|s,n)$$

where the prior distribution probability p(s, n) represents the likelihood of any other noise in the ECG signal. Thus, the objective function of the variational mode is expressed in Equation (6) as follows:

(6) $$Obj(s,n)=R_{error}+R$$

where Rerror is the reconstruction error and R is the regularization, which is the smoothness of the sparsity of noise. Thus, in Equations (7) and (8),

(7) $$R_{error}=||x(t)-\sum_{i=1}^{M}S_{i}(t)+n(t)||2$$

(8) $$R=\sum_{i=1}^{M}\lambda_{i}||s_{i}\left(t\right)\left|\left|p+\gamma \right|\right|n\left(t\right)||q$$

where $\lambda_{i}$ and γ are the regularization parameters and $\left\|.\right\|p$ and $\left\|.\right\|q$ are the norms. The model parameters are adjusted based on the input signal at the decomposition stage at a learning rate based on the observed data; thus, s_i_^(t)^ and n(t) parameters are iteratively updated to reduce any possible impact of noise and the parameters were achieved using Equations (9) and (10).

(9) $$s_{i}^{\left(k+1\right)}=s_{i}^{\left(k\right)}-\eta \frac{\delta (Obj)}{\delta .s_{i}}$$

(10) $$n^{k+1}=n^{k}-\eta \frac{\delta (Obj)}{\delta .n}$$

where η and k are the learning rate and iteration, respectively.

Though mode decomposition aids in feature extraction, the study went a step further to extract features using the R-peak, which aids in computing the Heart Rate Variability (HRV), RR interval (Time between successive R-wave peaks), and wave characteristics Q, S, and T in terms of amplitude and duration. The approach to compute the HRV is based on the standard deviation of the R-R intervals, which is measured using the Standard Deviation of NN intervals-SDNN). Also, by using the Root Mean Square of Successive Differences (RMSSD), the continual differences in the interval are computed. HRV metrics capture the SDNN and RMSSD of the extracted features, which are expressed using Equations (11) and (12):

(11) $$sdnn=\sqrt{\frac{1}{N-1}\sum_{i=1}^{N}{\left({RR}_{i}-mean\left(RR\right)\right)}^{2}}$$

(12) $$rmssd=\sqrt{\frac{1}{N-1}\sum_{i=1}^{N-1}{\left({RR}_{i+1}-mean\left({RR}_{i}\right)\right)}^{2}}$$

Three types of features are imperative in feature extraction, namely, time-domain, frequency-domain, and time–frequency domain. The time-domain feature $f_{\left(time\right)}$ like the mean, standard deviation, root mean square (RMS), skewness, and kurtosis from each segment were computed.

The discrete Fourier transform (DFT) signal is used to compute the spectrum of finite duration signal expressed in Equation (13) by

(13) $$X[k]=\sum_{n=0}^{N-1}x\left[n\right].e^{-j\frac{2\pi kn}{N}}$$

where *X[k]* and *N* are the DFT coefficient at index *k* and total number of samples, respectively. *x[n]* is the discrete time-domain signal. Then, to recover the original discrete signal, the Inverse Discrete Fourier Transform is expressed in Equation (14) by

(14) $$x\left[n\right]=\frac{1}{N}\sum_{k=0}^{N-1}X\left[k\right].e^{j\frac{2\pi kn}{N}}$$

where *x[n]* and *X[k]* are the n-th time domain in a sample and the k-th frequency domain components, respectively. N represents the number of points in the sequence and *j* is the imaginary units.

Furthermore, the time–frequency domain features $f_{\left(timefreq\right)}$, which addresses the changes in frequency over time, was computed using a Short-Time Fourier Transform (STFT) that maps subsequent segments of ECG signal into dimensions of time and frequency expressed in Equation (15), as follows:

(15) $$X\left(t,f\right)=\int_{-\infty }^{\infty }x\left(\tau \right)w\left(\tau -t\right).e^{-j2\pi f\tau }d\tau$$

where w(τ−t) is the window function positioned at *t.*

Finally, the fusion of multiple domains $f_{fused}$ is expressed to concatenate all the different domain features into a single vector representation that can be expressed in Equation (16) as:

(16) $$f_{\left(fused\right)}=\left[f_{\left(time\right)}\right|f_{\left(freq\right)}|f_{\left(timefreq\right)}]$$

where $f_{\left(fused\right)}$ is the fused vector, $f_{\left(time\right)}$ is the vector of the time-domain feature, $f_{\left(freq\right)}$ is the frequency-domain feature, and $f_{\left(timefreq\right)}$ is the vector of time–frequency domain features. 

### 3.4. Statistical Analysis

Zero-crossing rate (ZCR) calculates the number of times the preprocessed signal crosses zero within a time *t* window. ZCR for the continuous-time signal is expressed in Equation (17) as

(17) $$ZCR=\frac{1}{T}\int_{0}^{T}\left|\frac{dS_{f}\left(x\left(t\right)\right)}{dt}\right|dt$$

where *S_f_* represents the sign function of *x(t)* between (+1, 0, −1) in Equation (18), as follows:

(18) $$x\left(t\right)=\left\{\begin{array}{c} +1,\;for\;x\left(t\right)>0,\\ -1,\;for\;x\left(t\right)<0\\ 0,\;if\;x\left(t\right)=0 \end{array}\right.$$

### 3.5. Bio-Inspired Key Generation

The bio-inspired method was inspired by the concept of the half-life of a radioactive substance, which was considered in the formulation of the kestrel-based search algorithm [55]. The half-life was expressed as having *N* unstable substances that decay at time ***t*** is expressed in Equation (19):

(19) $$\frac{dN}{dt}=-\gamma N,$$

Which can be simplified in Equations (20)–(22), as follows:

(20) $$\gamma_{t}=\gamma_{o}{.e}^{-\varphi t}$$

(21) $$\varphi =\frac{\ln0.5}{-t_{\frac{1}{2}}}$$

(22) $$if\varphi \to \left\{\begin{array}{c} \varphi >1,\;trail\;is\;new\;\\ \;0,\;otherwise\; \end{array}\right.$$

where φ is the decay constant and t½ is the period of half-life representing the required time for $\gamma_{t}$ to become half of $\gamma_{o}$. γ is the light intensity variation generated at random intervals between [0, 1]. The bio-inspired method has been applied in several problem domains and the selection of the parameters in the method was demonstrated through an experiment with promising performance results [55,56,57]. Among the parameters include the flight (0.8) and perch (0.2) modes. The initial population in the bio-inspired algorithm is generated using Equation (23), as follows:

(23) $$\gamma_{t}=[\gamma_{1},\gamma_{2},\ldots ,\gamma_{n},RandBit()]$$

*Randbit*() represents a random bit generator to ensure randomization and reduce the chance of unauthorized breaking of the encryption key. A unique key is generated from the vectorized fused feature and hashed. Afterward, the bio-inspired algorithm final key is generated using the following Equation (24):

(24) $$Fkey=[UniqueFeatureKey,\gamma_{t}]$$

(25) $$UniqueFeatureKey=(F_{1},F_{2},\ldots F_{n})$$

where $F_{i}$ is the fused multi-domain vectorized ECG feature. The randomness of the generated key with Shannon entropy and Min-entropy for the bio-key generated on the ECG signal of the subject were evaluated to assess randomness through entropy in Equation (26):

(26) $$Shannonentropy=-\sum_{i}P_{i}{log}_{2}P_{i}$$

(27) $$Minentropy=-\log_{2}\left(\right)$$

The final key Fkey is used with Chacha20 for encrypting ECG bio-signals. The encryption scheme is mathematically modeled such that it takes the plaintext P*_i_* and applies the XOR on the keystream k to output the cypher text *C_i_* at each position *i* as expressed by Equation (28):

(28) $$C_{i}=P_{i}⊗K_{i}$$

**Figure 1:**
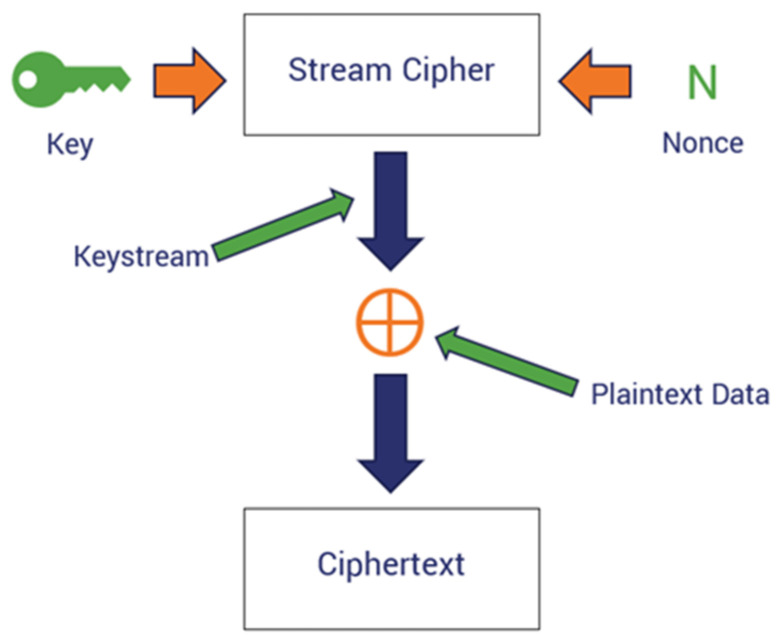
Diagram of the encryption scheme.

Furthermore, the decryption scheme is then expressed by Equation (29), as follows:

(29) $$P_{i}=C_{i}⊗K_{i}$$

where $P_{i}$ represents the plaintext and $C_{i}$ is the cipher text at the ith position. The keystreams *k* are extracted from the fused vector representation. Figure 1 below illustrates the encryption scheme.

### 3.6. Algorithm to Implement Lightweight Encryption

The algorithm to implement the lightweight encryption steps is expressed with Algorithms 1–3. Algorithm 1 indicates the steps in ECG signal processing. In this algorithm, raw ECG signal is inputted, and then different mathematical computations are performed to output the pre-processed ECG signal.
**Algorithm 1:** ECG signal Preprocessing1.**Input:** raw ECG signal, $F_{min}$, $F_{max}$,2.**Compute:** $H\left(f\right)$ using Equation (1)3.**Compute:** $h\left(t\right)$ using Equation (2)4.**Compute:** $F_{norm}$ using Equation (3)5.**Output:** Preprocessed ECG signal 

Algorithm 2 presents the steps to implement the feature extraction.
**Algorithm 2:** Feature Extraction
*//data-adaptive variational model decomposition*1.**Compute:** *x(t) using Equation (4)*2.**Compute:** Obj(s,n) *using Equation (6) to compute the objective function and likelihood of error in mode reconstruction*3.**Compute:** sdnn,  rmssd4.**Compute:** discrete Fourier transform (DFT) signal using Equation (11)5.**Compute:** $X\left(t,f\right)$ using Equation (15)6.**Compute:** $f_{\left(fused\right)}$ using Equation (16)7.**Compute**: ZCR using Equation (17)8.**Output**: fused feature vector

Algorithm 3 presents a fused feature vector and the bio-inspired key generation steps.
**Algorithm 3:** Fused feature vector and bio-inspired key generation1.**Initialize population:** $F_{i}$2.**Generate** Unique key for the feature vector in the string representation3.**Generate**: random key using Half-life4.**Generate**: Fkey5.**Output**: Fkey6.**Apply:** Fkey with the Chacha20 encryption scheme as expressed by Equations (28) and (29).

## 4. Results

**Figure 2:**
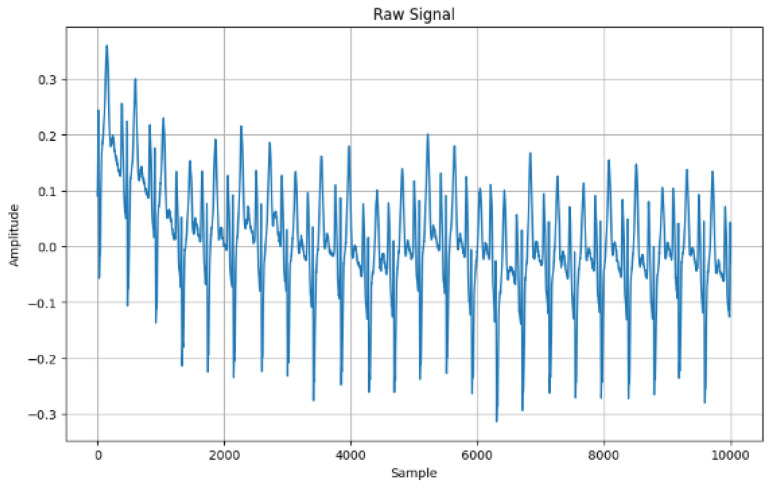
Raw signal.

This section presents the experimental results. Figure 2 shows the frequency of the original ECG data. The highest amplitude is a little above 0.3 and the lowest is below the −0.3 amplitude.

**Figure 3:**
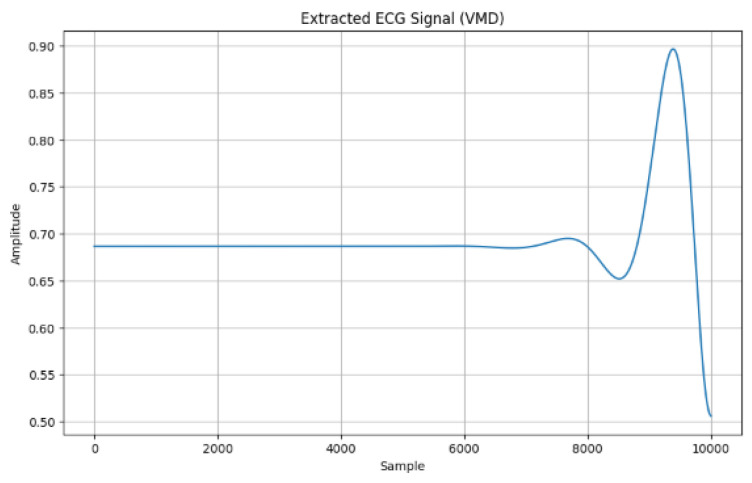
ECG signal extraction using adaptive VMD.

Figure 3 shows the extracted ECG signal using the adaptive VMD method in which the amplitude signal was 0.90, such as the peak for the 10,000 samples.

**Figure 4:**
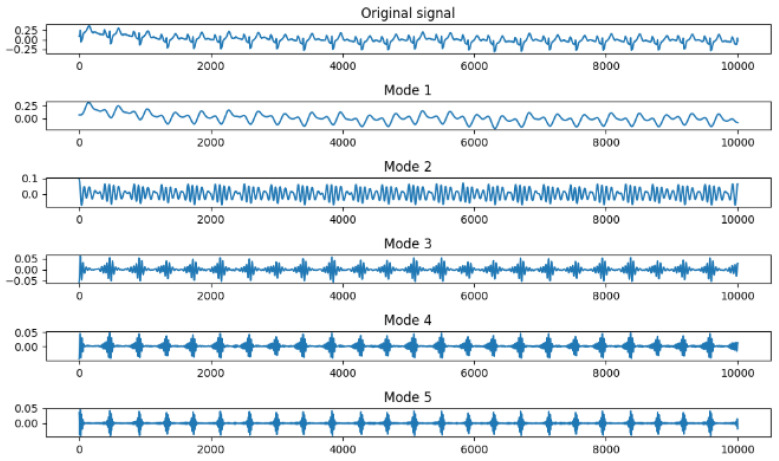
Five adaptive Variational Mode Decomposition (VMD).

Figure 4 shows the adaptive VMD consisting of three stages. The first phase of the decomposition initialized the noise tolerance value (0.0), in which the mode five decomposition is created to enable a view of the behavior of the frequencies. The bandwidth constraint of 2000 was set within a tolerant convergence criterion of 1 × 10^−7^. The second stage is the visualization of the mode decomposition in terms of the original signal and the five-mode decomposition as shown in Figure 4.

**Figure 5:**
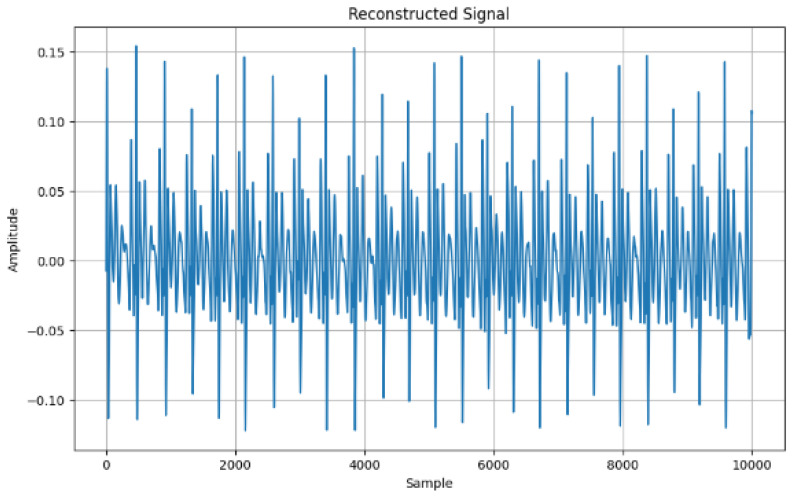
Signal reconstruction with adaptive VMD.

Finally, Figure 5 shows the mode reconstruction where all noise has been removed from the signal in preparation for feature extraction. The highest amplitude was a little above the 0.15 amplitude and the lowest amplitude was below −0.10.

**Figure 6:**
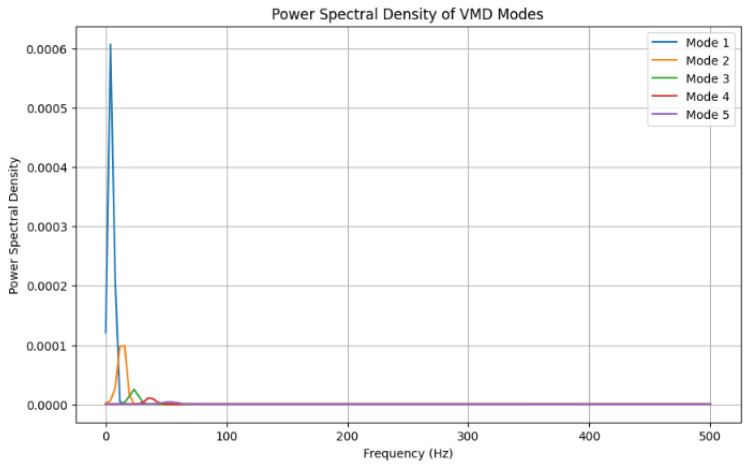
Power spectral density of adaptive Variational Mode Decomposition (VMD).

Figure 6 depicts the power spectral density of the adaptive VMD models where mode 1 (in Figure 5) happens to have the highest peak of (0.0006) and mode 2 (0.0001).

**Figure 7:**
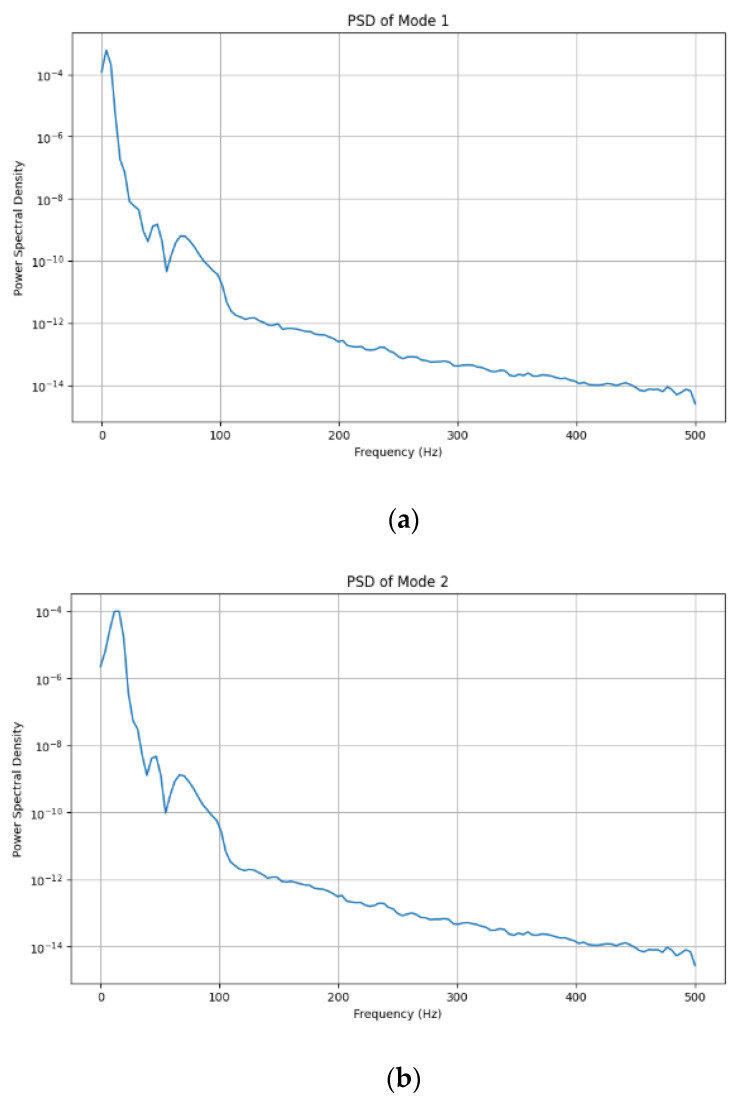

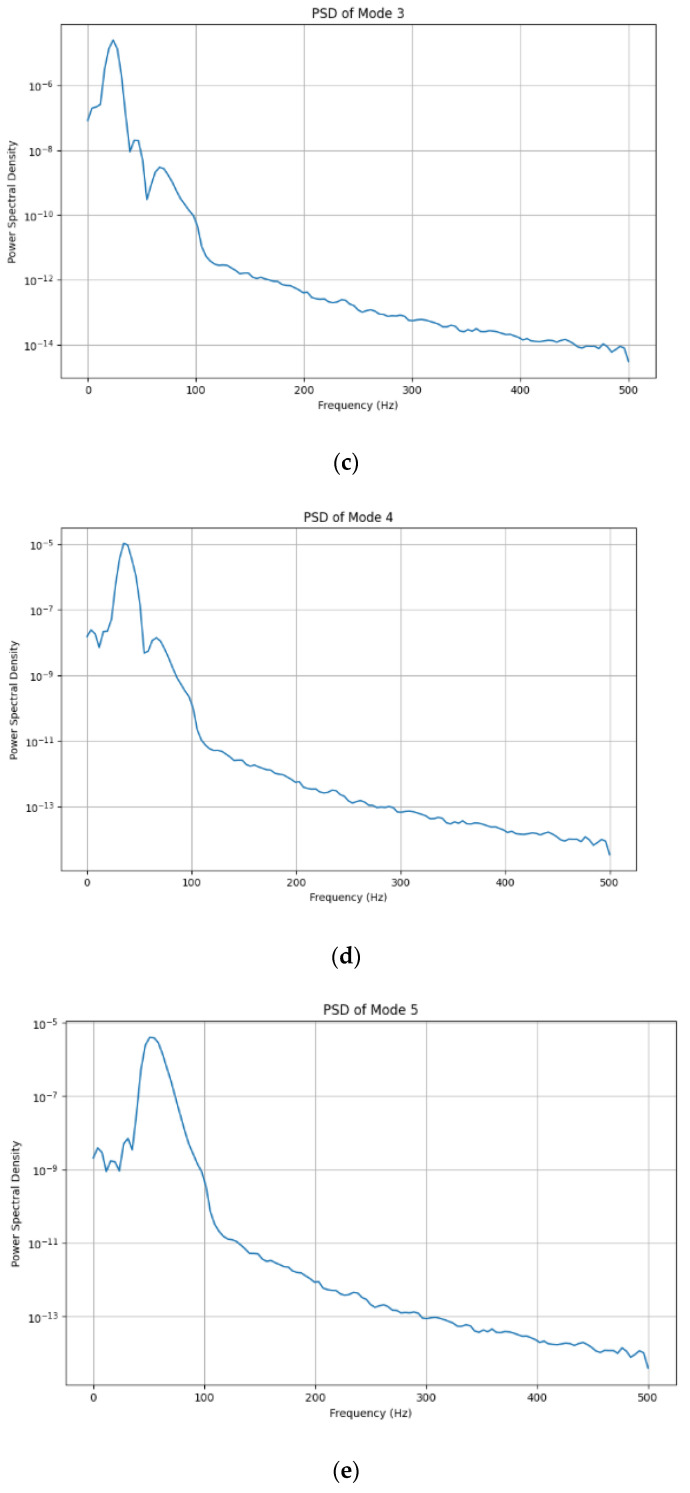

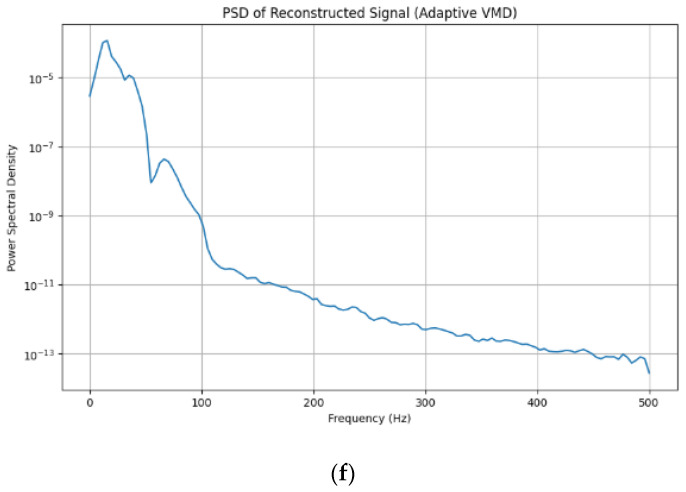
Power spectral density of the adaptive VMD with five modes.

Figure 7 shows each adaptive VMD mode for the reconstructed signal. The mode shows the decomposition of the ECG signal to help understand the frequencies in each segment of the mode.

**Figure 8:**
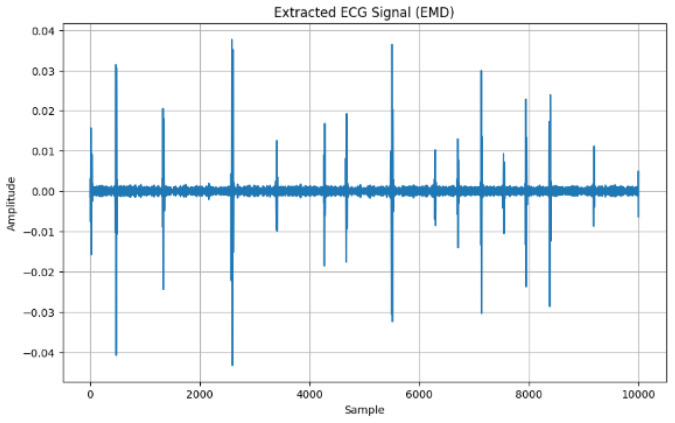
Extracted ECG signal with EMD.

Figure 8 shows the Empirical Mode Decomposition method (EMD) as another approach to recovering the ECG signal in which the first Intrinsic Mode Function (IMF) of the ECG signal is identified as it recognizes the highest frequency. IMF varies in amplitude and frequency, where the high amplitude is approximating to 0.04.

**Figure 9:**
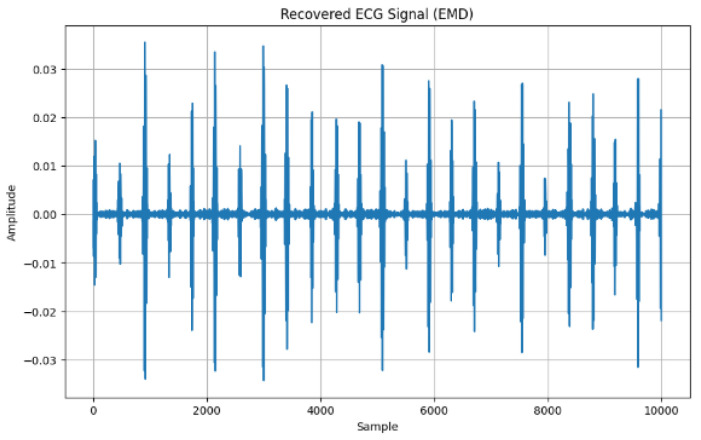
Recovered ECG signal (EMD).

Using the EMD approach, the first IMF becomes the recovered signal, which is depicted in Figure 9.

**Figure 10:**
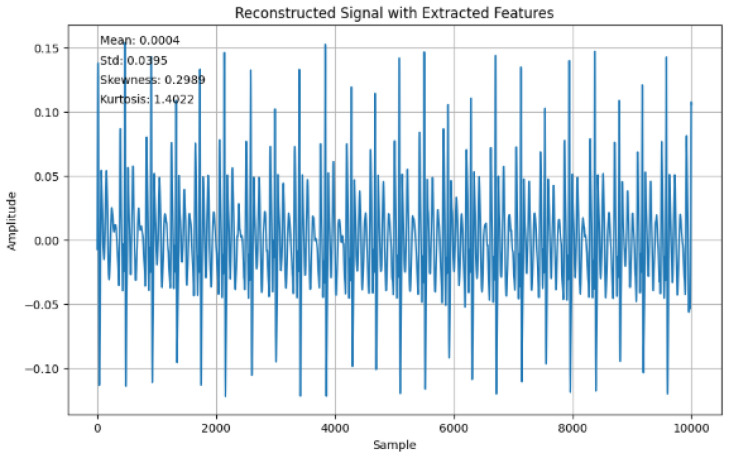
Reconstructed signal with extracted features.

Figure 10 shows the reconstructed signal with the time-domain features extracted showing the mean (0.0004), standard deviation (0.0395), skewness (0.2989), and kurtosis (1.4022).

**Figure 11:**
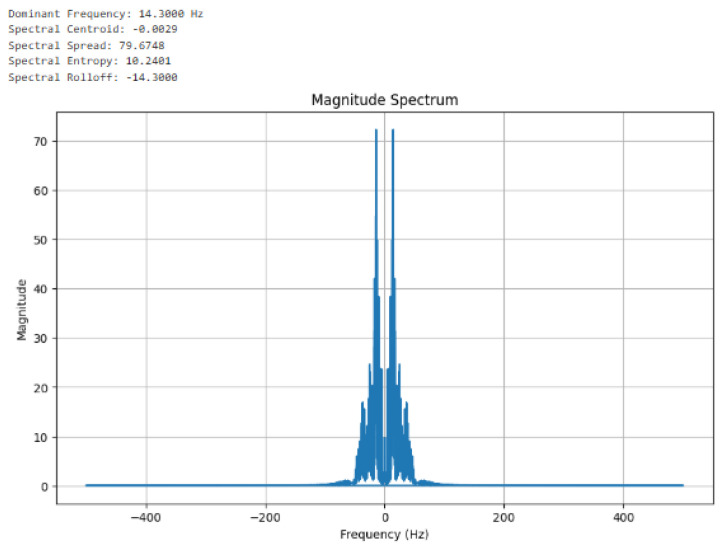
Frequency domain features extracted from the reconstructed signal using a continuous Fourier transform.

**Figure 12:**
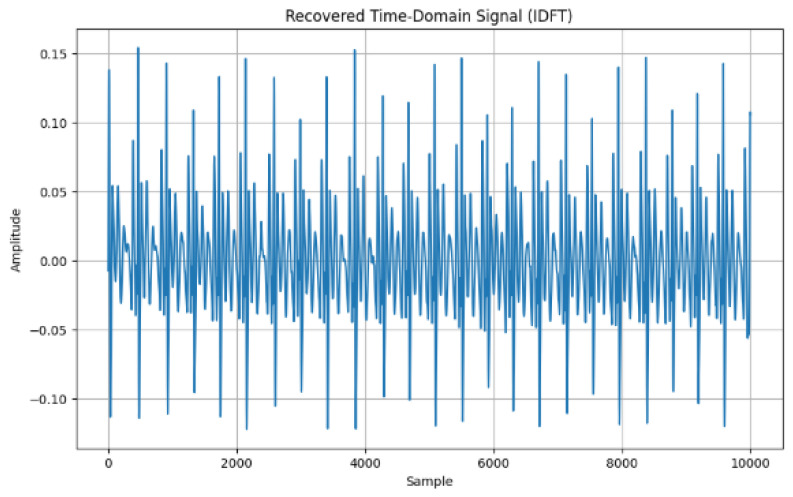
Time domain signal.

Figure 11 shows the frequency domain feature extractions using the continuous Fourier transform. The magnitude spectrum was computed and the features captured are the dominant frequency (14.3000 Hz), spectral centroid (−0.0030), spectral spread (80.3951), spectral entropy (10.1530), and spectral Rolloff (−14.0000). In Figure 12, the Inverse Discrete Fourier Transform (IDFT) is presented as it converts the frequency-domain features back to the corresponding time domain; such conversion confirms Figure 10 and Figure 11.

**Figure 13:**
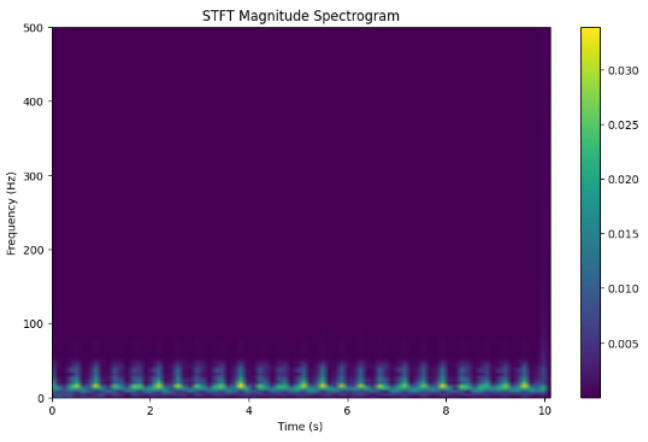
STFT Magnitude spectrogram visualization.

Figure 13 shows the STFT magnitude spectrogram showing the actual signals. It highlights the time–frequency property for an accurate representation of the signal. Colors represent the amplitude frequency at each point in time. Dark portions are regions with one signal. Through the distribution, the patterns can be visualized and timed in seconds (s) displayed.

**Figure 14:**
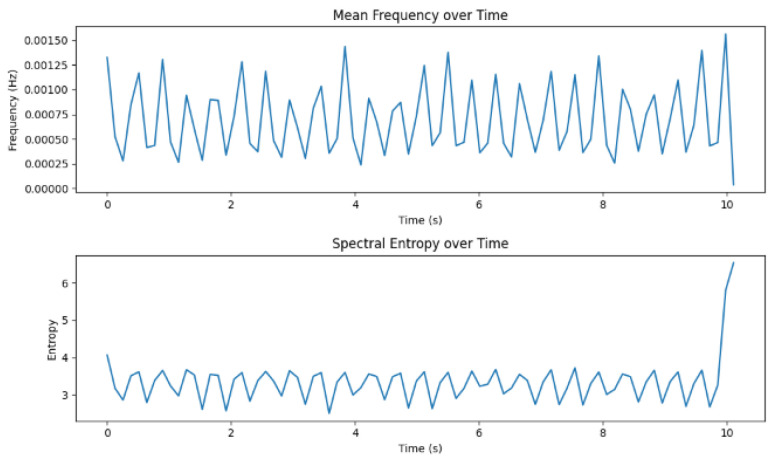
Frequency domain.

Figure 14 shows the frequency domain in terms of the mean and spectral entropy over time. The mean frequency describes the central frequency of the power spectrum concentration.

**Figure 15:**
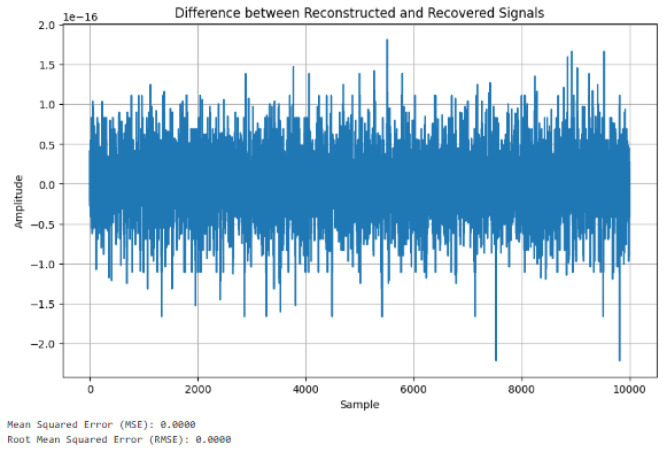
Differences between reconstructed and recovered signal.

**Figure 16:**
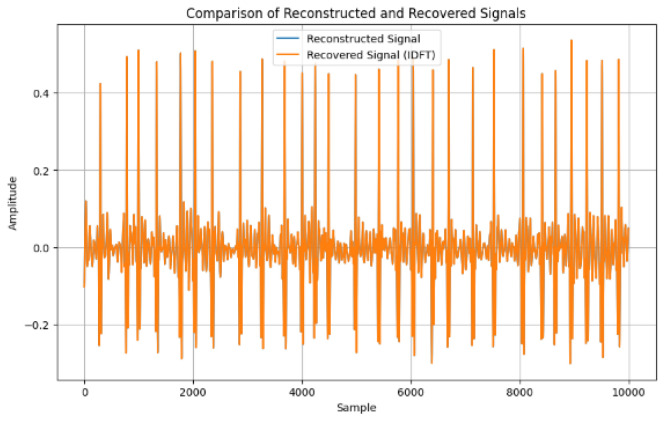
Comparison of reconstructed and recovered signal.

Figure 15 shows the difference between reconstructed and recovered signals. The MSE and RMSE are the statistical methods used to find the differences, which demonstrates that the means were both 0.00, suggesting that there is no variation between the reconstructed and the recovered signals. Figure 16 shows further analysis of the reconstructed and recovered signal, which further demonstrates no variation.

**Figure 17:**
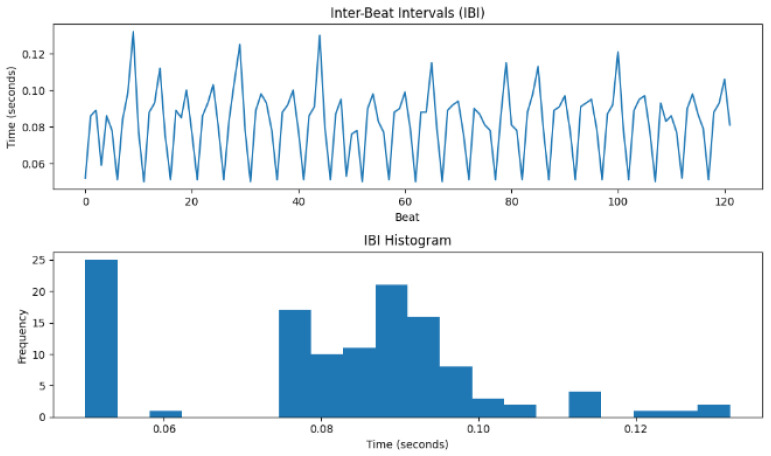
Inter-beat interval (IBI) and IBI histogram.

Figure 17 displays both the IBI and its histogram. The fluctuation in the heartbeat varies with time indicates a functioning nervous system of the individual. Again, the histogram demonstrates the time interval of successive heartbeats over some time. These features of the heartbeat are imperative in creating an effective encryption scheme from the human features.

The HRV computation utilized standard deviation among others to calculate, in beats per minute (BPM), the heart rate, which was recorded as 738.00 (BPM), RMSSD (0.0840 s), SDNN (0.0191 s), NN50 (0), and pNN50 (0.00%). Also, the zero-crossing rate was 0.0245. Table 1 shows the aggregated domain features in terms of the time domain and frequency domains.

**Figure 18:**
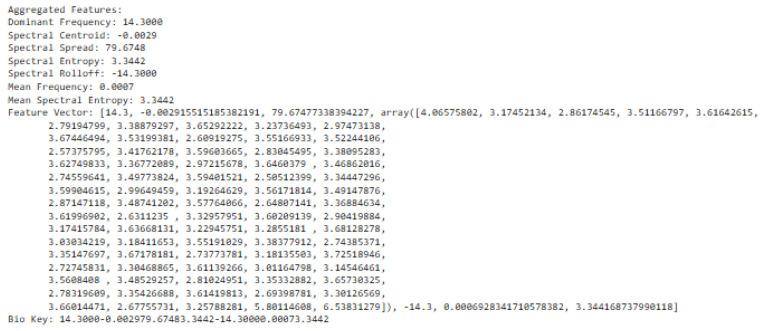
Fused feature vector with adaptive VMD.

The fusing approach is an aggregation of time–frequency domain features, frequency domain features, time-domain features, EMD, and adaptive VMD features, which were vectorized. Having extracted these features as shown in a vector representation, the bio-inspired algorithm was utilized to generate a random key. Before this, the extracted feature vector is converted to string representation and the bio-key was applied to finally generate the encryption key. The encryption scheme was evaluated by loading different ECG signals to extract the features in vector format and applied for ECG signal encryption. The resultant feature extracted is shown in Figure 18 as

**Figure 19:**
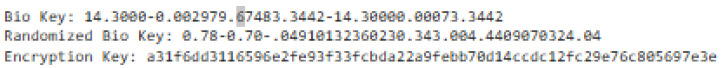
Key generation.

Figure 19 depicts the bio-key, random bio key, and encryption key obtained from the feature vector.

**Figure 20:**
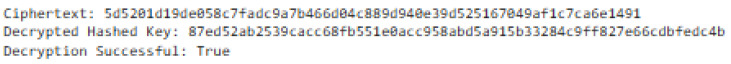
Cipher and decrypted text.

Figure 20 shows the cipher and decrypted text. It also indicated the status of decryption.

A large population of kestrels can cover a broader search area, which increases the chances of finding a global optimum key but requires more computational resources. In this study, the population size was 200, to limit the computational cost of devices. However, a small population might fail to explore enough of the solution space, leading to a less secure key. Table 2 provides a comparison of the execution time of the encryption algorithms.

The computing time (execution time) was considered in terms of time of encryption, time of decryption, and time for key generation. In this instance, the same population size in the bio-inspired search method was maintained and the efficiency was recorded in terms of execution time. The speed of encryption and decryption were measured (in microseconds (µs)) by executing the algorithm and recording the time taken. From Table 2, it can be observed that Chacha20 has a total execution time of 27 µs, Chacha (29.63 µs), and Salsa20 (30.90 µs). Thus, Chacha20 was efficient and suitable for resource-constrained devices.

The potential vulnerability of the proposed encryption scheme was the noise removal from the raw signal, which may impact the encryption key generation. Thus, the signal reconstruction approach was introduced to further remove noise, thus providing the surety and robustness of the encryption scheme.

## 5. Discussion

This study focused on extracting features from ECG signals to create an encryption scheme. The results demonstrated the capability to extract features into vector forms using time domain, frequency domain, and time–frequency domains. The advantage of time-domain approaches is their simplicity, which is based on statistical measures such as mean and variance. Again, this suggests less computation in signal analysis. Furthermore, it is effective for stationary signal analysis due to the statistical properties that do not change over time [58]. The advantage of the frequency domain is that it is more applicable for non-stationary signals due to the changing frequency over time. Again, it is more useful for processing more compact signals. Furthermore, it helps to identify noise signals and filter the signal to identify the most relevant signals [59]. Whereas, the advantage of time–frequency domain is that it provides a more comprehensive analysis of the signal, in both time and frequency domain features, over time. Again, it shows more patterns and insight into the signal. Bao, Yan [12] provided fusing mechanisms that leverage Variational Mode Decomposition (VMD) and Convolutional Neural Networks (CNNs) for feature extraction toward user identification system development. The VMD model can decompose features and remove noise that may affect the ECG signal quality. Modes from these decomposed features were derived (Figure 5) after all residual noise had been removed to enable feature extraction. In furtherance to this, the Empirical Mode Decomposition method (EMD) is an approach to enable the ECG signal to be recovered using the IMF to find the highest frequency. The EMD technique is used to assist in understanding the frequency oscillations of heartbeats.

In terms of the time domain, by using the reconstructed signal features, the mean (0.0004), standard deviation (0.0391), skewness (0.1562), and kurtosis (1.2205) were all extracted (Figure 10) from the ECG data. The use of IDFT and DFT further validated the consistency of the conversion as captured in Figure 10 and Figure 11, such that the frequency-domain features are correlated with the back with the time-domain representation features.

With respect to the frequency domain, the mean and spectral entropy were also displayed in Figure 14. The spectral entropy helps quantify the uncertainty that might occur in the power distribution of the signal from different frequency points of view. While high spectral entropy signifies the complexity of frequency distribution, the contrary suggests a more predictable distribution. Moreover, the power distribution is indicative of how power can be dispersed over frequencies [28].

Using metrics of MSE and RMSE to measure variation, Figure 15 shows that there was no deviation between the reconstructed and the recovered signals. Hence, the viability of our methods is demonstrated.

The features of the heartbeat are critical in the process of developing unique bio-keys for encryption; these features are indicated in the inter-beat interval and histogram as illustrated in Figure 17. Metrics about the time and frequency domain were extracted through HRV computation as outlined in Table 1 from these HRV-related features.

In the final analysis, the study utilized the feature extraction methods in this paper to aggregate these metrics into feature vectors to enable the encryption scheme development. Notably, HRV was employed as a simple key-generation approach [8]. Moreover, our approach provides a more robust key generation approach because it leverages the capabilities of more feature extraction approaches to create a more complex feature vector.

Bio-inspired algorithms, which by their nature provide randomness, increase the robustness of the encryption key. The bio-inspired algorithm Kestrel’s initial population is randomly generated as potential solutions to be found. This randomized searching enables bits of the fused feature vector to be chosen at random. The concept of half-life, introduced in the Kestrel algorithm, provides an additional layer of randomization, via the light intensity variation in the half-life component, in the formulation of the encryption key.

The selection of parameters in bio-inspired algorithms is crucial for the efficiency and security of the encryption key generation. Tuning the population size of the bio-inspired algorithm optimizes the generation of robust unpredictable keys that enhance the security of encryption systems. Moreover, an improper parameter selection may result in weak keys or longer computation times, which could undermine the effectiveness of an encryption system. Setting an optimal population size can provide an efficient key generation for an encryption algorithm and also ensure security. The trade-off between security and efficiency depends on the computing limitations and the strength of the encryption key [40]. Thus, while a smaller population size may guarantee faster results, it may compromise the security of the generated key. On the other hand, a larger population size provides a more secure encryption key at a high computational cost. Thus, while Table 2 provides the trade-offs on executing time as the measure of efficiency, Chacha20 was efficient.

This simplified encryption key is provided as a parameter for use in the ChaCha20 encryption algorithm while The ChaCha20 algorithm is a symmetric-key algorithm as it ensures that the same key is both used for encryption and decryption in applications that require high speed and security [41]. Our study has provided an encryption scheme that is suitable for high-speed environments and for IoMT devices that can process data quickly but also ensure the security transmission of data using extracted human features. Our study extracts human features from the bio-signals of these IOMT devices to ensure that these bio-signals can be securely encrypted and transmitted. From a theoretical perspective, this study contributes to the introduction of the unique bio-inspired Kestrel algorithm with its randomized searching and its half-life component, both of which add layers of randomization for a more secure key for ECG signals. In this research, the impact of noise on generated encryption keys and the impact of bio-inspired search parameters on key generation were, respectively, addressed with signal reconstruction and the use of population tuning of the bio-inspired search method.

## 6. Conclusions

The paper sought to extract features from ECG signals to create an encryption scheme that does not only rely the on time domain, frequency domain, or time–frequency domains. Instead, through the use of the mode decomposition approach, features of an intra-relationship were extracted and statistically validated to help create a fusion feature vector. Through leveraging this vector with the unique features of the bio-inspired Kestrel algorithm, a more robust encryption scheme is provided. Thus, by leveraging the chacha20 encryption algorithm with our feature extraction and key generation approach, we provide an encryption scheme that might be suitable for lightweight devices like IoMT. Future work includes further evaluation of this proposed encryption scheme within the context of IOMT devices in the real-world environment.

## Figures and Tables

**Table 1:** Domain features.

Aggregated Features	Value	Type of Feature Domain
Dominant Frequency:	14.3000	Frequency domain
Spectral Centroid:	−0.0029	Frequency domain
Spectral Spread:	79.6748	Frequency domain
Spectral Entropy:	10.2401	Frequency domain
Spectral Rolloff:	−14.3000	Frequency domain
Mean Frequency:	0.0007	Time–frequency
Mean Spectral Entropy:	3.3442	Time–frequency
Mean	0.0004	Time domain
standard deviation	0.0395	Time domain
Skewness	0.2989	Time domain
Kurtosis	1.4022	Time domain

**Table 2:** Comparison of execution time.

Encryption Scheme	Encryption Time	Decryption Time	Key Generation Time	Total Time
ChaCha20	9.40 µs	9.75 µs	7.85 µs	27 µs
ChaCha	10.80 µs	10.98 µs	7.85 µs	29.63 µs
Salsa20	11.45 µs	11.60 µs	7.85 µs	30.90 µs

## Data Availability

Data are available upon request.
